# The Effect of Nonpharmaceutical Interventions Implemented in Response to the COVID-19 Pandemic on Seasonal Respiratory Syncytial Virus: Analysis of Google Trends Data

**DOI:** 10.2196/42781

**Published:** 2022-12-21

**Authors:** Hersh D Ravkin, Elad Yom-Tov, Lior Nesher

**Affiliations:** 1 Faculty of Health Sciences Ben-Gurion University of the Negev Beer Sheva Israel; 2 Microsoft Research Herzeliya Israel; 3 Faculty of Industrial Engineering and Management Technion Haifa Israel; 4 Infectious Disease Institute Soroka University Medical Center Beer Sheva Israel

**Keywords:** RSV, respiratory syncytial virus, search engine, Google Trends, Google, respiratory, children, pharmaceutical, intervention, COVID-19, pandemic, virus, infection, health

## Abstract

**Background:**

Respiratory syncytial virus (RSV) is a major cause of respiratory infection in children. Despite usually following a consistent seasonal pattern, the 2020-2021 RSV season in many countries was delayed and changed in magnitude.

**Objective:**

This study aimed to test if these changes can be attributed to nonpharmaceutical interventions (NPIs) instituted around the world to combat SARS-CoV-2.

**Methods:**

We used the internet search volume for RSV, as obtained from Google Trends, as a proxy to investigate these abnormalities.

**Results:**

Our analysis shows a breakdown of the usual correlation between peak latency and magnitude during the year of the pandemic. Analyzing latency and magnitude separately, we found that the changes therein are associated with implemented NPIs. Among several important interventions, NPIs affecting population mobility are shown to be particularly relevant to RSV incidence.

**Conclusions:**

The 2020-2021 RSV season served as a natural experiment to test NPIs that are likely to restrict RSV spread, and our findings can be used to guide health authorities to possible interventions.

## Introduction

Respiratory syncytial virus (RSV) is the leading cause of lower respiratory tract infection in children worldwide. Preterm gestation and several other underlying conditions particularly increase the risk of hospitalization and severe disease [1]. Although no specific treatment exists, prophylactic administration of monoclonal antibodies, when timed correctly, mitigates some of the risks. The American Academy of Pediatrics guidelines limit the duration of treatment with monoclonal antibodies to 5 months, with maximal benefit derived when treatment is initiated prior to the onset of the local RSV season [1]. Seasonality—including the start, peak, and end weeks—in RSV has been studied extensively and generally follows a set pattern within each country, with little variation from year to year. For the start week, even relatively major variations, when they rarely occur, do not exceed 1 month [2]. This regularity is key for proper timing of prophylaxis administration [1]. Beyond timing, a consistent spatiotemporal pattern of RSV epidemics has been established in previous years [3]. As there is no known animal reservoir of human RSV, transmission occurs solely through close contact with other humans [4]. Changes in human behavior, therefore, are likely integral to the dynamics and seasonality of RSV epidemics.

Nonpharmaceutical interventions (NPIs) are policy-based strategies used to mitigate the effects of infectious diseases. When vaccines are unavailable, NPIs are the primary recourse for reducing transmission rate and decreasing the burden on health care systems. NPIs may be grossly categorized as personal, communal, or environmental [5]. While the latter 2 may be reasonably implemented across an entire population, it is difficult to enforce adherence to personal NPIs in very young children. This is a particularly important consideration in RSV, where young children are the primary at-risk group.

Surveillance of RSV outbreaks is not uniformly rigorous across the world [6]. Changes in health-seeking behaviors and viral surveillance during the COVID-19 pandemic further complicate the interpretation of epidemiological data [7]. However, previous research has shown that the volume of search engine queries can serve as a proxy for the incidence of respiratory diseases [8-11]. Initial attempts to harness internet search data to monitor viral incidence were shown to be naïve. For instance, Google Flu Trends, a system that predicted the influenza load from the Google search volume for specific terms, was shown to overestimate these loads [12]. However, work since then has improved the models that predict loads of influenza-like illness from these data [13,14]. In the case of RSV, the Google query volume for the term “RSV” has been demonstrated to be a good proxy for RSV incidence [15]. This correlation has been used to draw conclusions regarding the dynamics of RSV transmission when epidemiological data are insufficient [15,16].

In 2020, countries around the world instituted various NPIs to combat the COVID-19 pandemic [17]. Researchers have reported that the 2020-2021 RSV season was exceptional, in that its peak was both delayed and changed in magnitude [16,18-20]. Here, we re-establish the correlation between RSV incidence and internet search data and use the latter as a proxy to investigate the association of the various NPIs instituted in response to the COVID-19 pandemic with the abnormalities of the 2020-2021 RSV season.

## Methods

### Data Sources

Nonsentinel observational data on RSV incidence were obtained from the European Centre for Disease Prevention and Control’s Surveillance Atlas of Infectious Diseases, an interactive tool that pools data collected from its member states through the European Surveillance System [21]. Data included in the study range from week 40 of 2014 to week 42 of 2021.

Search query volume data were gathered from Google Trends using the Google Trends Anchor Bank package [22]. Search query volume data for the Google Trends topic “Respiratory syncytial virus” were gathered for the period between week 9 of 2016 and week 43 of 2021.

**Figure 1:**
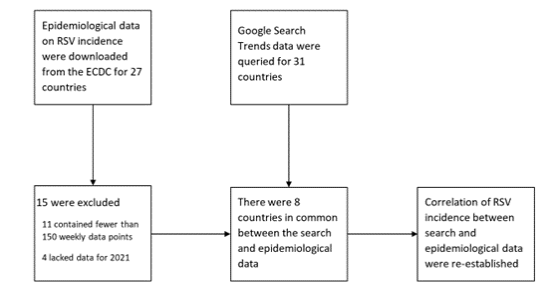
Data sources and process used to validate correlation between RSV incidence and the internet search volume for RSV. ECDC: European Centre for Disease Prevention and Control; RSV: respiratory syncytial virus.

**Figure 2:**
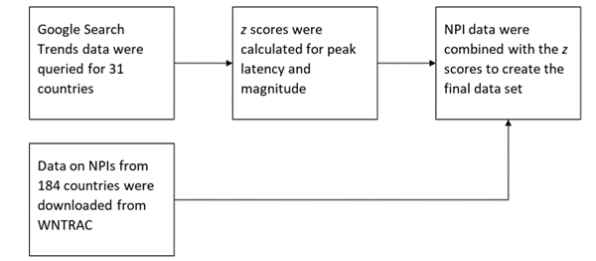
Data sources and processing used to generate the final data set. NPI: nonpharmaceutical intervention; WNTRAC: Worldwide Non-pharmaceutical Interventions Tracker for COVID-19.

Data on NPIs were taken from Worldwide Non-pharmaceutical Interventions Tracker for COVID-19 (WNTRAC). Briefly, WNTRAC is a comprehensive data set consisting of over 7000 NPIs implemented worldwide since the start of the COVID-19 pandemic. WNTRAC includes NPIs implemented in countries across the world, classifying them into a taxonomy of 16 NPI categories. NPI events are automatically extracted daily from Wikipedia articles using natural language processing techniques and are manually validated to ensure accuracy and veracity [17]. WNTRAC data up to December 17, 2021, are included in this study. Figures 1 and 2 provide a schematic overview of the data collection and processing.

### Preprocessing of RSV Incidence Data

The original European Centre for Disease Prevention and Control data included 27 countries. Of these, 11 countries were excluded because they contained fewer than 150 weekly data points. An additional 4 countries were excluded because they contained no data for 2021, leaving a matrix of epidemiological data for 12 countries available for confirming the correlation between epidemiological and search trends data.

Weekly search trend data were collected for 31 countries using Google Trends Anchor Bank. Only countries with sufficient Google Trends data were included in the study. An effort was made to include countries from both hemispheres and various continents to improve generalizability. Data were smoothed to remove noise by calculating a rolling average with a 5-week moving window. Annual peaks were identified by calculating the local maxima of each country’s weekly RSV incidence using a minimum horizontal distance of 35 samples between adjacent peaks (ie, a minimum of 35 weeks had to be present between adjacent peaks; this was achieved with the scipy.signal.find_peaks function, distance=35). Of the 31 countries for which search data were gathered, 8 were also present in the epidemiological data and could be used in the correlation analysis.

To allow comparison of the deviation from average of peak latency (ie, the extent to which the peak was delayed) and magnitude among different countries, standard scores were calculated for each in accordance with the function *Z = (x – μ) / σ*, where *Z* is the standard score, *x* is the observed peak week or peak magnitude for 2021, *μ* is the mean peak per week divided by the magnitude as calculated on the basis of prior years included in the study, and *σ* is the SD value of the peak week divided by the magnitude as calculated on the basis of prior years included in the study. These standard scores represent the 2 target features (ie, outcome variables) used in the study. 

### Preprocessing of NPI Data

NPI data for the countries included in the study were obtained from WNTRAC. Several NPI types were recategorized to make them amenable to representation in a tabular format; namely, NPIs with unique values (eg, specific countries from which there were travel restrictions) and restrictions on mass gatherings. The NPIs with unique features were reorganized as either “some” or “all” based on whether they referred to restrictions pertaining to specific countries or blanket restrictions on all countries, respectively. Restrictions on mass gatherings, which originally displayed a specific numerical limit, were binned, grouping restrictions on gatherings of 10 or less, 10-100, 100-250, 250-500, and ≥500 persons. The NPI “changes in prison-related policies” was removed as it was instituted in very few countries and because symptomatic RSV predominantly affects young children. Additionally, the NPIs “declared state of emergency” and “contact tracing for COVID-19 patients” were deemed unrelated to the dynamics of the RSV outbreak and were therefore removed from the data set to decrease the effect of multicollinearity. NPI subtypes “other” and “na” were combined and recategorized as “unspecified” in the interest of interpretability, with no subsequent change in performance.

Rather than considering all NPIs instituted throughout the study period, NPIs included in the final matrix were limited only to those interventions instituted during the 3 months preceding the expected (average calculated over the past years included in the study) peak week.

### Statistical Analysis

Spearman correlation analysis was used to calculate the correlation between epidemiological and search data. Correlation between peak magnitude and latency was determined by fitting a linear regression model.

Linear regression models were fitted to each of the two main target variables, peak latency and peak magnitude, after a subset of highly performing features was chosen through sequential backward selection. Backward selection was implemented using the scikit-learn SequentialFeatureSelector with default cross-validation and *R*^2^ scoring. Relative contribution of the various NPIs to each of the outcomes was evaluated by calculating their feature importance using the SHAP (Shapley Additive Explanations) package (version 0.39.0), one of the most robust approaches currently available for explaining machine learning outputs [23]. The SHAP package’s LinearExplainer was used to account for the correlation among various NPIs. For general pipeline development and validation, scikit-learn (version 0.22.1) was used. All analysis was conducted in Python (version 3.7.7).

## Results

### Overview

**Figure 3:**
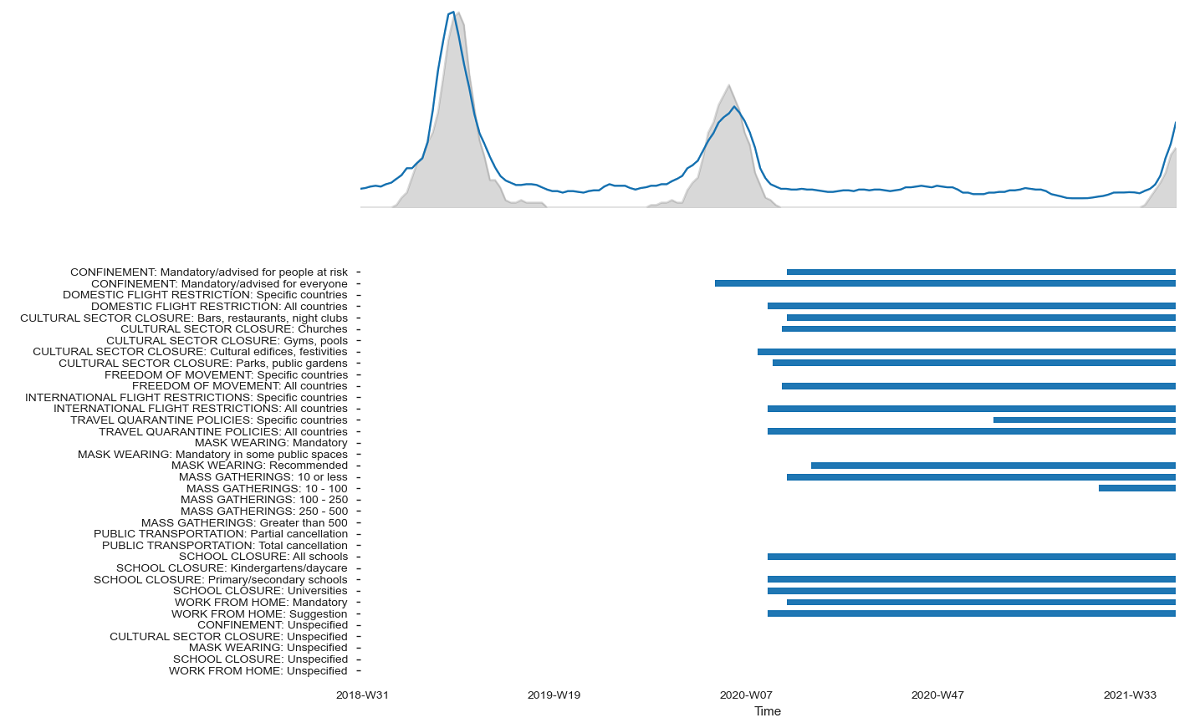
Changes in timing of respiratory syncytial virus incidence relative to the implementation of nonpharmaceutical interventions during the 2020-2021 season in Germany.

We first re-established the correlation between RSV case incidence and Google query volume for RSV, which was then used to infer RSV incidence for the 31 countries in our study. We then used a regression model to predict the normalized peak latency and the peak magnitude of RSV incidence in each country during the 2020-2021 season. Relative contribution of the various NPIs to each of the outcomes was then estimated using Shapley values. An example of the timing between the NPIs included in the study and the spread of RSV during the 2021-2022 season in one of the countries (Germany) is shown in Figure 3.

### Validating the Correlation Between Search and Epidemiological Data

**Figure 4:**
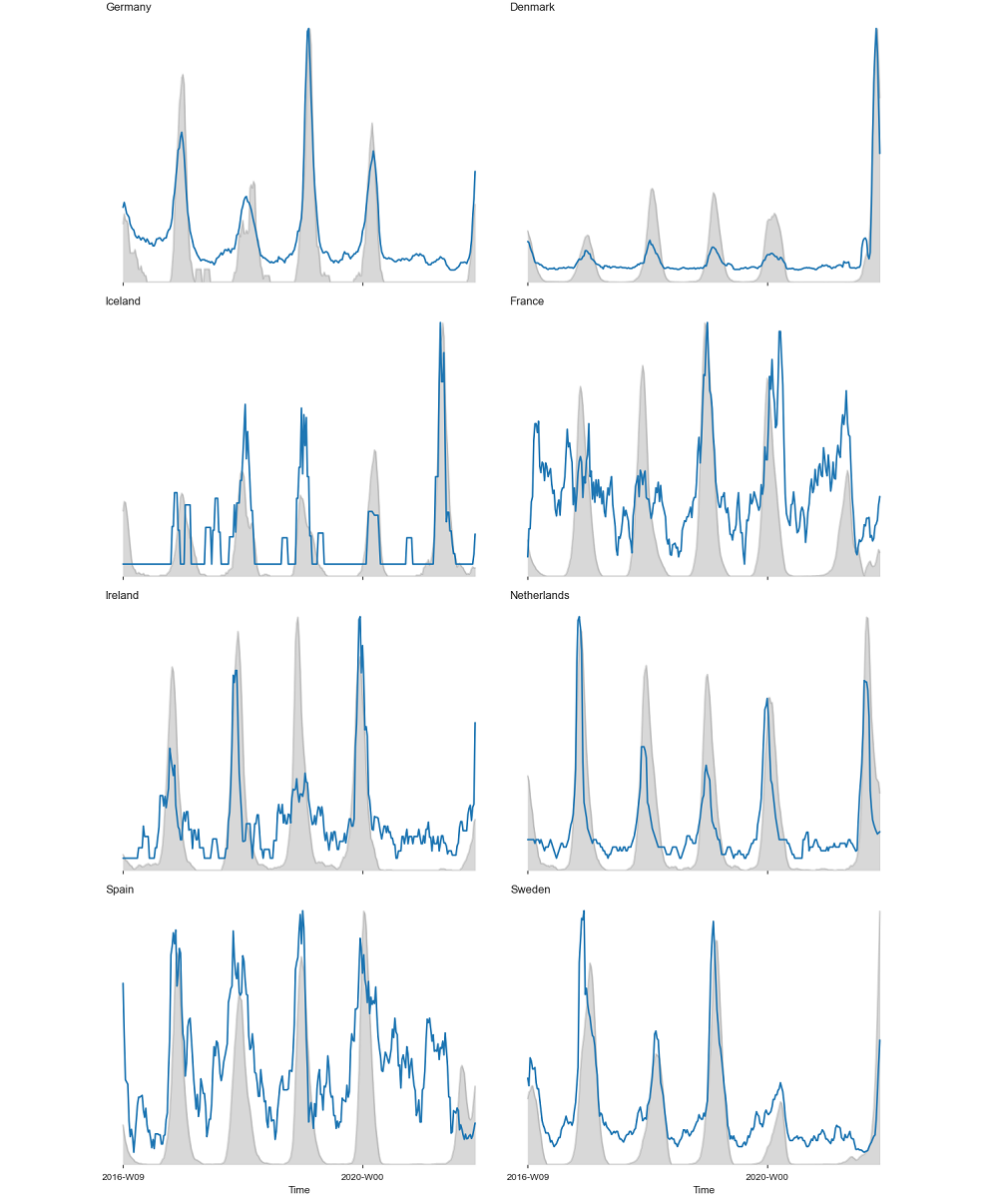
Correlation of epidemiological and search data in countries for which both were available.

The correlation of epidemiological incidence data on RSV with internet search volume in countries for which both were available was, on average, 0.61 (n=8 countries, estimated over a period of 291 weeks; Figure 4), validating past research and suggesting that search data could indeed be used as a proxy for RSV incidence during the 2020-2021 season. This correlation is displayed graphically in Figure 3, which shows that changes in search trends closely follow the epidemiological data. The gray shaded area represents epidemiological data on RSV incidence; the blue line depicts the search data volume.

### Correlation Between Peak Latency and Magnitude

The rank regression model of peak latency as a function of country and peak magnitude for the years preceding 2021 was significant (*R*^2^=0.13, *P*<.001), with country not shown as a significant explanatory variable. Applying this model to the year of the COVID-19 pandemic yielded an *R*^2^ of 0.07 (*P*=.07). This shows that the correlation between RSV peak latency and magnitude broke down during the 2020-2021 RSV season.

### Evaluation of Linear Regression Models

Two target variables, peak latency and peak magnitude, were examined in this study. For each, a subset of the most indicative attributes was chosen using backward stepwise selection. A linear regression model was fit to each of the target variables using each set of the chosen attributes. Both regression models were significant (adjusted *R*^2^=0.815, *F*_19,11_=7.967, *P*<.001) and (adjusted *R*^2^=0.799, *F*_19,11_=7.261, *P*<.001) for peak latency and peak magnitude, respectively.

### Insights From the Linear Regression Models

**Figure 5:**
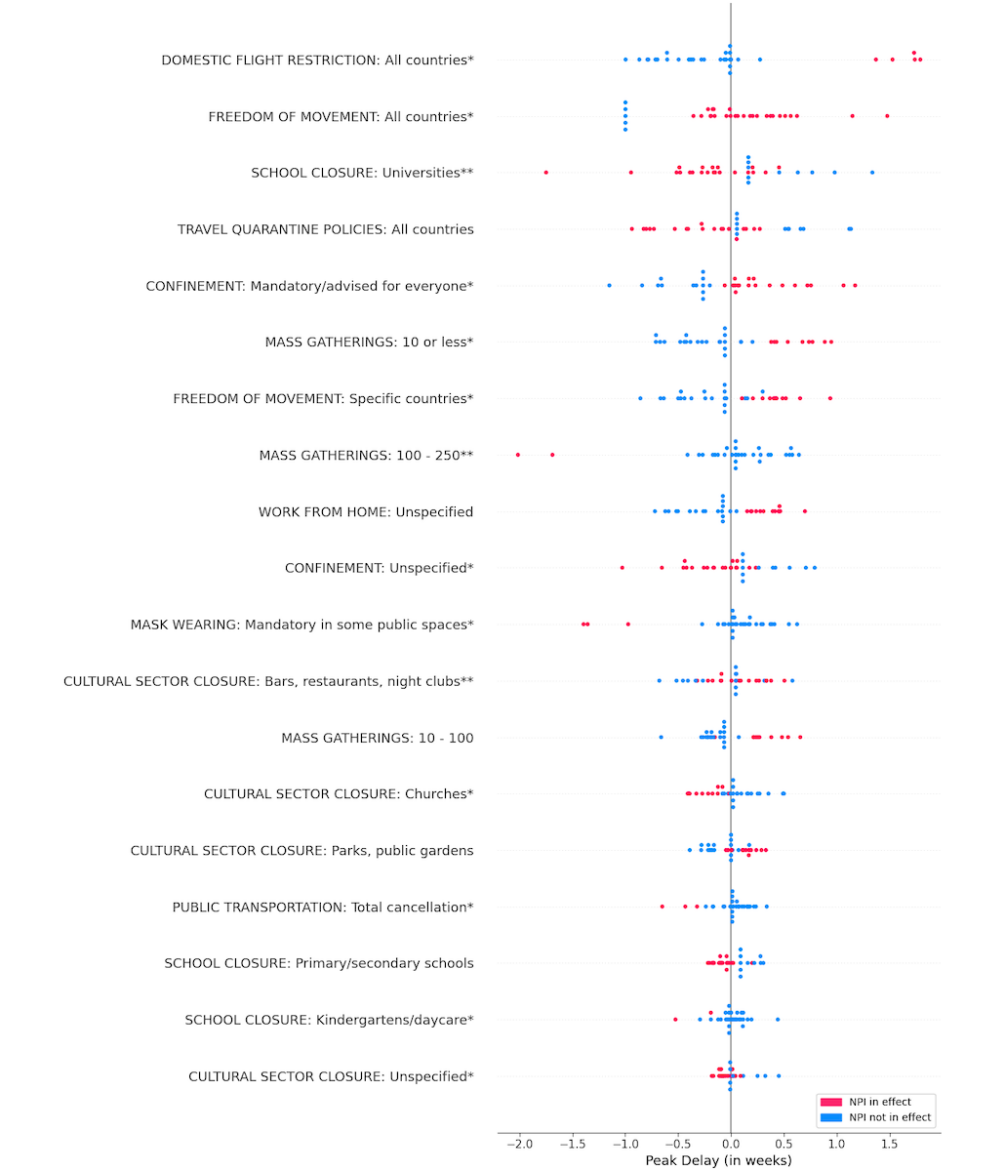
SHAP (Shapley Additive Explanations) summary plot for peak latency. Statistical significance: *P<.05, **P<.001. NPI: nonpharmaceutical intervention.

**Figure 6:**
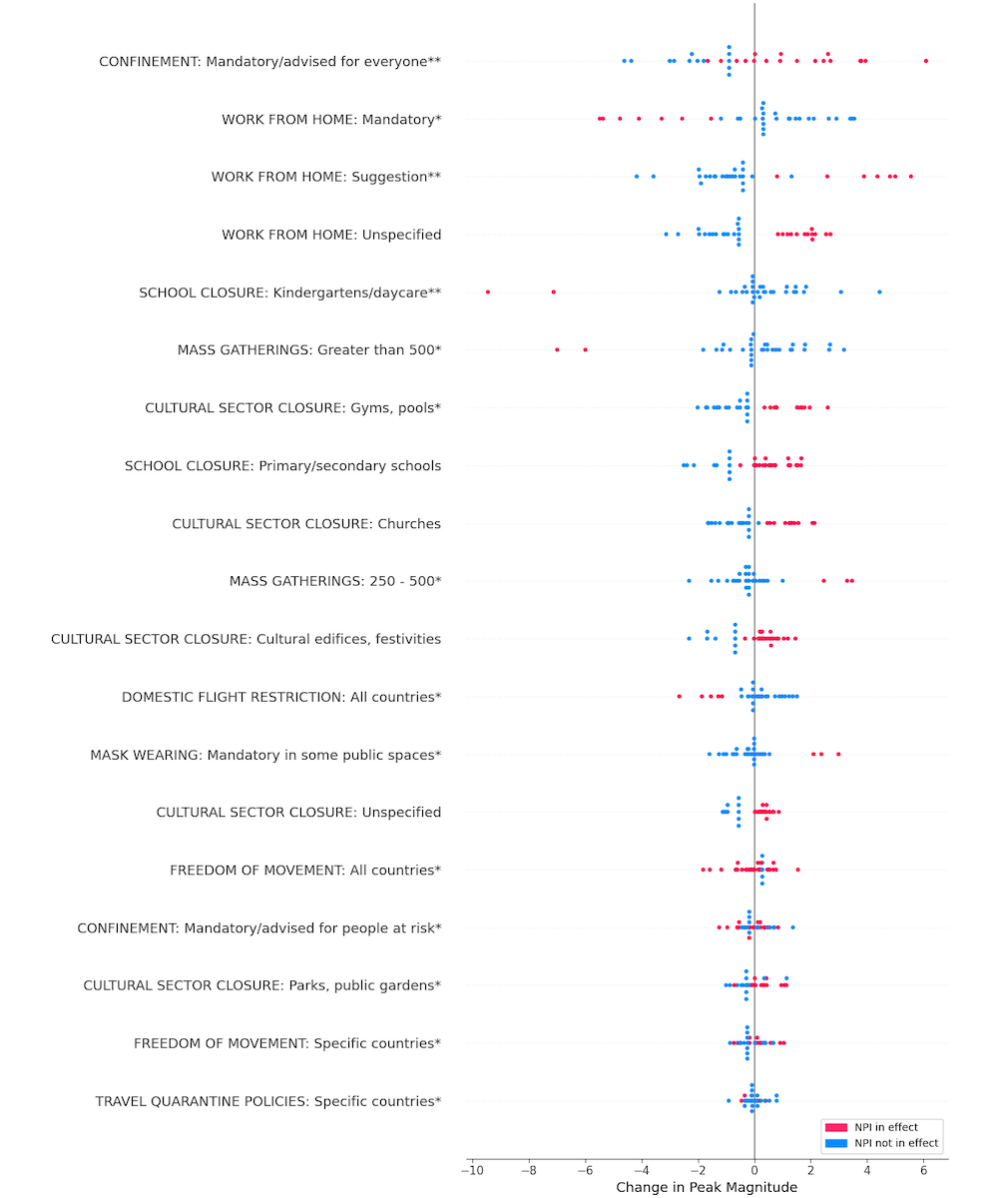
SHAP (Shapley Additive Explanations) summary plot for peak magnitude. Statistical significance: *P<.05, **P<.001. NPI: nonpharmaceutical intervention.

We analyzed the contribution of different NPIs to each of the two target variables. Multivariate feature importance (SHAP value) was calculated for all features in each of the two final models. Figures 5 and 6 highlight the effect of the various NPIs on peak latency and peak magnitude, respectively. Each point on the plot represents a country in the study; the color of the point indicates whether the NPI was in effect. For each feature, horizontal position relative to the midline (ie, expected value) indicates its contribution to model output. Features are arranged in descending order of mean absolute importance. It is important to note that all countries in the study experienced delays in the RSV peak. Leftward dispersion of data points in the peak latency plot, therefore, does not suggest that a feature caused an earlier peak than usual, but rather that it had a relatively lower association with the peak delay.

## Discussion

### Principal Findings

Our results support the hypothesis that NPIs instituted to combat the COVID-19 pandemic are strongly associated with changes in both the latency and magnitude of peak RSV incidence observed during the 2020-2021 RSV season. Owing to the lack of effective pharmacological treatment or vaccines for COVID-19, the global response to the pandemic relied mainly on the institution of NPIs. Various countries instituted disparate measures at different times [17]. The differential impact of these strategies has been posited to be partially responsible for the changes observed in the dynamics of respiratory viruses other than COVID-19 in 2021 [24]. Our study analyzed RSV incidence based on internet search volume and official epidemiological data reported by various agencies and used data analysis techniques to model the effect of NPIs on RSV incidence.

Linear regression analysis of peak magnitude as a function of peak latency demonstrated a significant correlation between peak latency and magnitude for the years preceding the COVID-19 pandemic. This correlation, however, broke down during the pandemic. Furthermore, linear regression analyses modeling peak latency and peak magnitude as a function of instituted NPIs identified distinct influential features for each target feature.

### Google Search Trends as a Proxy for RSV Incidence

Several studies have validated the efficacy of Google Search Trends query volume as an accurate proxy for RSV incidence [15,16]. We re-established the validity of these findings for the 2020-2021 RSV season. In keeping with previous studies, our analysis shows that changes in internet search data from most countries closely paralleled the observed epidemiological changes [15]. It is worth noting that while a recent study of global RSV seasonality included data on 27 countries, data for many of the countries had to be extracted from various national databases rather than being available from official RSV surveillance programs [2]. Although larger studies exist, these rely on data collected from published literature [25]. Surveillance data are particularly limited in middle- and low-income countries [2]. Although the need for increased epidemiological surveillance is undeniable, our study collected data on 31 countries in a manner that could potentially be automated and used for epidemiological decision-making in real time.

### Correlation Between Peak Latency and Magnitude

We found that, prior to the pandemic, the yearly timing of the RSV peak was linearly correlated with its magnitude. This linear correlation was disrupted during the pandemic. Our results substantiate the hypothesis that the institution of various NPIs accounts for a high degree of variation in the timing and magnitude of the RSV outbreaks experienced by various countries.

### Effects of Specific NPIs

A group of interventions related to reduced population mobility was associated with both a delayed RSV peak and a reduced peak magnitude. Among these were both domestic flight restrictions and restrictions on international arrivals from all countries, although the effects of domestic flight restrictions were more unequivocal. Restrictions on international arrivals from selected countries also showed a significant association with delayed and reduced peaks. These findings may lend credence to the hypothesis that the consistent spatiotemporal patterns of RSV spread are indeed linked to population mobility and human behavior. In the United States, for instance, yearly RSV activity begins in Florida during November and ends in February-March in the upper Midwestern United States [3]. A recent study identified the same spatiotemporal pattern, albeit shifted, in the out-of-season RSV epidemics during the 2020-2021 RSV season. The same study also suggested that increased volume of domestic air travel, which coincided with the out-of-season peaks in their study, may have been responsible [16]. Interestingly, mandatory quarantine for all arriving travelers was not significantly associated with changes in peak latency or magnitude, while mandatory quarantine on arrivals from select countries had a significant but unclear effect on peak magnitude.

Closure of school at the kindergarten or daycare level led to a significantly reduced peak magnitude. This corroborates what is known about RSV transmission in this high-risk age group. Other NPIs, particularly personal NPIs such as mask-wearing and social distancing, are extremely difficult to implement for this demographic. Reopening of schools (at all age groups) was significantly associated with an increased risk for RSV recurrence in another study that did not consider different age groups separately [26]. In our study, however, school closure at other age levels was not associated with a reduction in peak magnitude. This may be due to the greater ability to implement mask-wearing and other NPIs while maintaining school attendance at these ages. Additionally, it is possible that relaxation of school closure regulations had an unintended opposite effect of reducing social distancing in these groups.

Limiting mass gatherings to 10 people or fewer, effectively restricting interactions to the size of 1 or 2 nuclear families, was significantly associated with delayed peak. This intervention is widely considered one of the most drastic and effective measures for preventing the spread of respiratory viruses. A similar effect was demonstrated in western Washington in February 2019, where extremely high snowfall led to citywide social isolation at the level of individual household units. Researchers found that such a high-intensity intervention instituted close to the onset of an epidemic had the predominant effect of delaying the peak, while initiation of NPIs at the height of epidemic intensity predominantly decreased the peak’s magnitude. In the case of RSV, which was at the height of its peak during their intervention, a 95% decrease in incidence was recorded [27]. Restricting mass gatherings to 10-100 people had the same effect; interestingly, restricting mass gatherings to 250-500 people was significantly associated with both reduced peak delays and a higher peak magnitude. This suggests that intervention at this threshold is less effective. Restriction of mass gatherings at the level of 500 people was associated with reduced peak magnitude, possibly echoing a similar conclusion to that of reduced population mobility. Restriction of mass gatherings at higher thresholds is also more difficult to interpret owing to greater variation in the types of gatherings. For instance, high-risk gatherings such as weddings, concerts, or clubs likely have different effects from lower-risk gatherings such as professional conferences or outdoor events.

### Limitations

As with any study involving predictive modeling without incorporating dedicated experimental variation, associations identified here cannot be used to infer causal insights without further study. However, NPIs that were implemented to handle the COVID-19 pandemic influenced RSV incidence, as we have shown. Thus, institution of these NPIs should be considered a natural experiment from which a causal effect can be inferred. Furthermore, the high granularity of the NPIs used in this study, while vastly improving model robustness, makes it more difficult to draw conclusive insights regarding the differential efficacy of NPIs. For instance, there are several instances in which more granular interventions, such as advised confinement for at-risk people or freedom of movement restrictions for specific countries, had a more significant effect on delaying peak incidence than their broader alternatives. While this most likely reflects the pattern adopted by many countries, of initially instituting more specific restrictions and gradually broadening them to include a greater segment of the population, it is difficult to substantiate this without conducting further research. Furthermore, some collinearity exists among the NPIs—for example, it stands to reason that countries with higher COVID-19 caseloads would implement a greater number of NPIs in parallel—thus increasing the multicollinearity of the data. We calculated the SHAP values presented using correlation-dependent feature perturbation to mitigate the effect of this collinearity to the greatest extent possible. Our study also did not consider the effects of climate, which is universally considered a significant factor contributing to RSV incidence.

### Conclusions

Successfully anticipating the timing of RSV outbreaks is crucial for maximizing the prophylaxis of at-risk neonates. Current American Academy of Pediatrics guidelines recommend prophylactic treatment of at-risk neonates with monoclonal antibodies. The efficacy of this treatment is limited, however, and timely administration of the prophylactic drug before the yearly RSV outbreak is key to maximizing outcomes. Beyond the timing of prophylaxis, concerns over the timing of the 2020-2021 RSV epidemic have led governmental agencies to express concerns over concomitant viral outbreaks exceeding the capacity of health care systems [28]. This highlights the need for an efficient framework to predict changes in RSV seasonality in real time.

Identifying which interventions have the most pronounced effects on attenuating RSV outbreaks is important not only to further the understanding of RSV dynamics, but also as a tool for decision-making in future viral outbreaks. While further research is needed, we believe that our work may be a stepping stone on the path to accumulating sufficient literature and expertise to begin incorporating internet search trends as surrogate data for viral surveillance. By providing additional evidence to support the role of population mobility and human behavior on both spatial and temporal elements of RSV spread, we believe we have also shed light on the viral dynamics of RSV.

## Abbreviations

NPI: nonpharmaceutical intervention
RSV: respiratory syncytial virus
SHAP: Shapley Additive Explanations
WNTRAC: Worldwide Non-pharmaceutical Interventions Tracker for COVID-19
